# Monitoring the dynamic vulnerability of an Arctic subsistence food system to climate change: The case of Ulukhaktok, NT

**DOI:** 10.1371/journal.pone.0258048

**Published:** 2021-09-29

**Authors:** Angus W. Naylor, James D. Ford, Tristan Pearce, David Fawcett, Dylan Clark, James van Alstine

**Affiliations:** 1 Priestley International Centre for Climate, University of Leeds, Leeds, West Yorkshire, United Kingdom; 2 School of Earth and Environment, University of Leeds, Leeds, West Yorkshire, United Kingdom; 3 Department of Global and International Studies, University of Northern British Columbia, Prince George, British Columbia, Canada; 4 Canadian Institute for Climate Choices, Vancouver, British Columbia, Canada; Swedish University of Agricultural Sciences and Swedish Institute for the Marine Environment, University of Gothenburg, SWEDEN

## Abstract

Vulnerability to climate change is highly dynamic, varying between and within communities over different timescales. This paper draws upon complex adaptive systems thinking to develop an approach for capturing, understanding, and monitoring climate vulnerability in a case study from northern Canada, focusing on Inuit food systems. In the community of Ulukhaktok, Northwest Territories, we followed 10 hunters over a 2-year period, asking them to document their harvesting activities and discuss their lived experience of harvesting under changing environmental and societal conditions. GPS monitoring and participatory mapping sessions were used to document 23,996km of trails (*n* = 409), with conversational bi-weekly semi-structured interviews and secondary instrumental weather data used to contextualise climate change within a nexus of other socioeconomic, cultural, and political stressors that also affect harvesting. Our results demonstrate that climate change has considerable potential to affect harvesting activities, particularly when its impacts manifest as anomalous/extreme events. However, climate change impacts are not necessarily the most salient issues affecting harvesting on a day-to-day basis. Instead, factors relating to economics (particularly financial capital and the wage-based economy), social networks, and institutions are found to have a greater influence, either as standalone factors with cascading effects or when acting synchronously to augment the impacts of environmental change.

## Introduction

The impacts of climate change have been observable in the Arctic for over four decades [1, 2]. Yet with current rates of warming in the circumpolar north at two to three times the global annual average, the incidence and severity of these adverse impacts is projected to increase [3]. Climate-induced environmental change holds especially severe implications for Inuit in Northern Canada, whose subsistence-focused livelihoods are closely linked to environmental conditions [4, 5]. It is for these reasons that many Inuit organisations and communities have identified climate change as a fundamental challenge to both their ways of life and human rights [4, 6, 7].

The challenges Inuit presently face when practicing subsistence extend beyond the simple biophysical impacts of climate change, however. Changing land, ice, and ocean environments are intersecting with a diversity of socially-constructed stressors—both dynamic and multiscale—that can affect food systems, food security, and circumpolar health [8–12]. For instance, previous studies have documented the role and importance of Inuit ecological knowledge in adaptation to climate change, finding that it underpins competency in harvesting and adaptation to changing conditions [13, 14]. Other research has examined the influence that top-down wildlife management practices, introduced in response to changing animal migration patterns, can have on harvesting, mental health, and wellbeing [15, 16]. A broad body of scholarship has also assessed how food systems altered by climate change are changing from a post-harvest perspective, in particular examining how cultural change, demography, and altering social norms are affecting sharing networks [17–19].

Despite an improved understanding that the impacts of climate change in the Arctic are dependent upon multiple socioeconomic, political, and cultural factors, little research has captured the interaction of these dynamics across space and time [20]. Much past research has been structured around the organising concept of ‘vulnerability’, which was developed within the disaster studies and natural hazards fields during the late 1970s through a focus on the ‘un-naturalness’ of disasters [21–23], and was subsequently incorporated into human dimensions of climate change scholarship [24–28]. Vulnerability research seeks to identify and understand the factors that put people and places at risk or reduce their ability to respond to threats. It is important to note that vulnerability approaches are designed to understand dynamics and drivers of change, and do not establish populations *a priori* as vulnerable; a common theme in much vulnerability research is instead an emphasis on adaptation, adaptive capacity, resilience, and agency [29, 30].

Past vulnerability research—in the Arctic and globally—has been critiqued for producing a temporally “static” account of vulnerability, despite conceptual thinking and approaches that highlight that the experience of vulnerability is highly a dynamic and transient process [e.g. 20, 31]. Other researchers have also criticised vulnerability approaches for privileging biophysical drivers over social phenomenon in their analyses [12, 32], for bringing a deficit framing to the study of climate change [33, 34], or for their propensity to produce an incomplete picture of vulnerability through a poor accounting of endogenous and exogenous drivers of differential risk [35, 36]. More recent critiques have also suggested that while vulnerability research is effective at signposting which stressors are present, such work infrequently discusses *how* they interact or their relative precedence [37]. Addressing these methodological and epistemological shortcomings represents a ‘grand challenge’ for vulnerability research, where new approaches are critically needed [29].

This paper examines how climatic and wider sociocultural, political and economic factors affect climate change vulnerability, focusing on Inuit hunters from Ulukhaktok, NT. The paper attempts to capture how these various factors interact over time, to develop an in-depth understanding of how climate change is being understood, experienced, and responded to. In doing so, our research aims to reconcile some of the aforementioned conceptual-methodological disconnects relating to past vulnerability assessments in the Arctic. We advance a new conceptualization of the Inuit subsistence food system, or ‘foodshed’, as a dynamic, complex adaptive system, and integrate this approach with the real-time monitoring of risk. We first outline the conceptual basis for our research approach. This is followed by an overview of the study area and documentation of the methods used. Our results and subsequent discussion highlight the spatial context of the subsistence-based foodshed, the relationships between place and the manifestations of vulnerability and adaptive capacity over time, and the role of climate versus other socially constructed factors affecting foodshed stability. While our approach is developed and operationalised in an Arctic context, it has global relevance for similar studies working with people whose lives and livelihoods are associated with diverse food environments.

## Methodology

### Conceptual approach

We draw upon, and advance, the heuristic approach for conceptualising climate vulnerability outlined in Naylor *et al*. [20] and apply it to a complex adaptive system: the subsistence foodshed of Ulukhaktok, NT. Derived from the principle of a ‘watershed’, a ‘foodshed’ refers to the social and physical landscape of a food system, within which foods are sourced, prepared, distributed or otherwise exchanged [38–40]. In the context of Ulukhaktok, the subsistence foodshed describes the physical extent of hunting, fishing, and foraging grounds on the lands, sea, and ice proximal to the community and the relationships and norms that govern how these foods are harvested and shared between its residents (see Study Area).

Studies addressing social-ecological phenomena are increasingly adopting complex adaptive systems theory [41–44]. A complex adaptive system (CAS) describes an set of components—self-organised and grouped relationally according to function—whose micro-interactions and non-linear interdependencies develop emergent, dynamic behaviours that can dictate whole-system characteristics [20, 42]. Crucially, CAS approaches place specific emphasis on the dimension of time within linked human-environment systems, and the ways through which time and a structure of a system can interact to develop emergent process-based phenomena [20]. Specifically, CAS theory argues that a system exists in a non-equilibrium state; that its components have the potential to disaggregate, develop webs of causality, and exhibit feedbacks, redundancy and adaptive learning; and that these can create hierarchical interactions that may be both endogenous and exogenous to the CAS in question [41, 43, 45, 46]. Framing system interactions as constantly changing and more than simple linear ‘cause and effect’ in this manner provides a particularly useful frame of reference for appraising and understanding the dynamic manifestations of vulnerability and risk that often emerge from climate-society interactions [20, 47].

**Fig 1:**
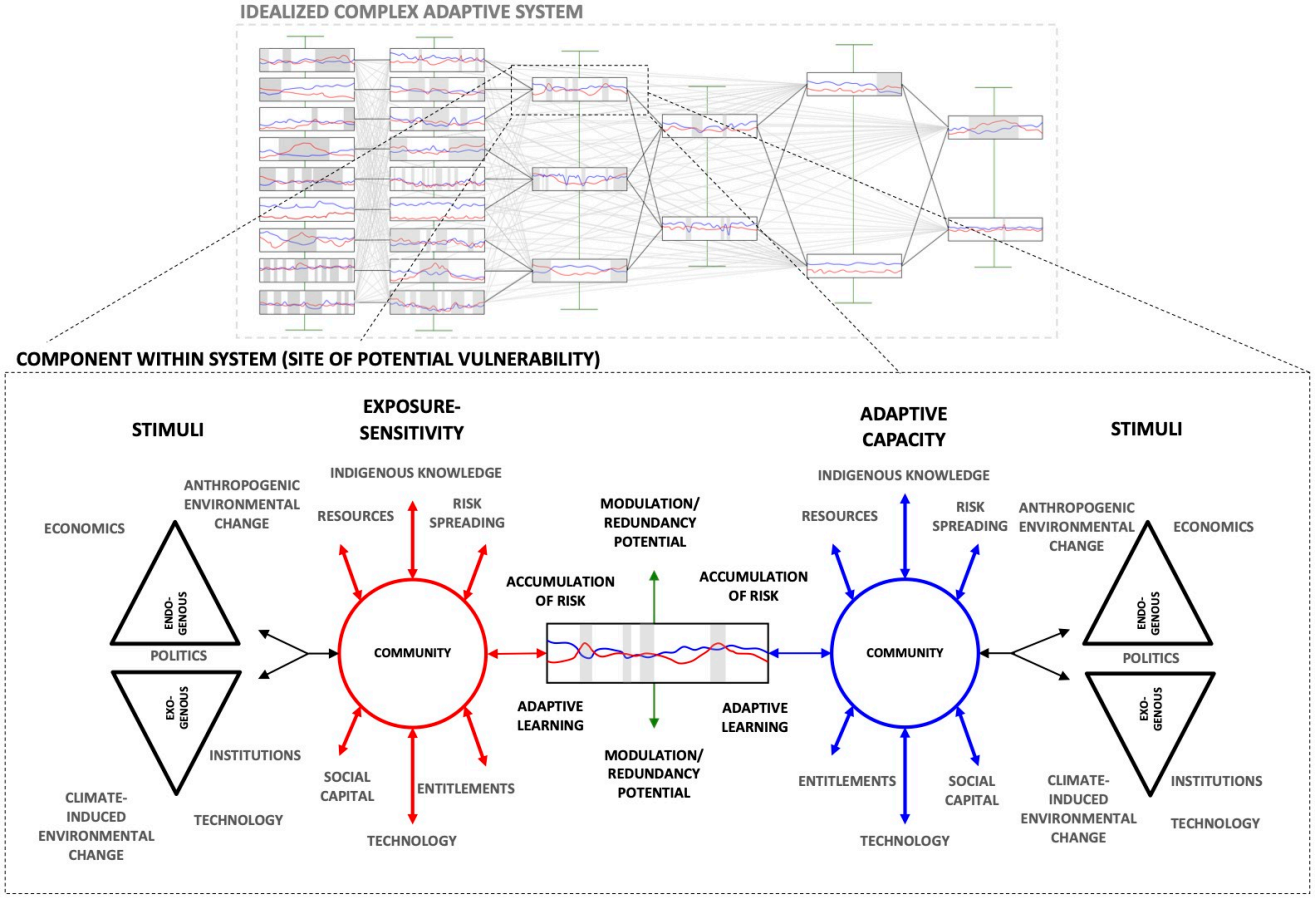
Idealized complex adaptive system, disaggregated into components according to their function. Here, potential vulnerability in each component of the food system (e.g. the availability of certain subsistence species, ability to access lands)—indicated by the box insert—is determined by the role of exogenous and endogenous stimuli and stressors, such as environmental change, the degree to which an individual has access to technology, or their access to entitlements. The ways in which these stimuli interact creates potential exposure-sensitivity (red) and adaptive capacity (blue) across time. The central ‘barcode’ illustrates this interaction and its dynamism, with the grey bars highlighting periods of adaptive deficit (vulnerability). Interconnectedness between components within the system allows vulnerability in a single component to affect or migrate to multiple other areas of the system through time, and to produce emergent/system wide changes (modified from Naylor *et al*. [20]).

However, in order to better understand the ways through which vulnerability might manifest or be ameliorated within a specific complex adaptive system, location-specific, geography of place research approaches are also essential [48, 49]. By compartmentalising the subsistence foodshed of Ulukhaktok, NT into components that can be appraised for their relative vulnerability in time and space, it is possible to identify transient or persistently at-risk dimensions within the system (e.g. access to hunting grounds, food storage capacity), and subsequently highlight these areas as priorities for adaptation (Fig 1). Moreover, by compartmentalising *but not detaching* components from their broader context in the system as a whole, a vulnerability approach based upon CASs theory allows for a stronger understanding of the influence of vulnerable components on total-system stability. In particular, the ways through which vulnerabilities might otherwise migrate or be contained as a result of stocks and flows between components and the development of emergent adaptive or maladaptive responses [46, 50].

In the context of the CAS vulnerability framing posited here, the potential susceptibility of components within a system (and by extension the system as a whole) is understood to be a function of the relative occurrence, duration, and magnitude of ‘exposure-sensitivity’ and ‘adaptive capacity’ [20, 26]. As outlined by Smit & Pilifosova [51] (see also Ford & Smit [52]), exposure-sensitivity describes the nature and rate at which stressors/stimuli (e.g. altered entitlements, climatic extremes, changes to social relationships) are applied, in addition to the pre-existing conditions that are present at their point of application. Adaptive capacity refers to the function of all relationships, expertise, and entitlements (and their ease of mobilization and utilization) that allow for the preparation, coping, or adjustment against stressors/stimuli throughout the duration of the latter’s application [26, 52]. A negative disparity between exposure-sensitivity and adaptive capacity creates an adaptive deficit (period of relative vulnerability), requiring either an increase in the strength of an adaptive response, or a change in the conditions developing of exposure-sensitivity [20] (Fig 1).

While there have been attempts to empirically ‘measure’ or provide values for magnitudes of adaptive capacity, exposure-sensitivity, and, therefore, vulnerability through the use of indicators and indices (e.g. [53, 54]), the efficacy of such approaches is contentious [32, 55]. This reflects the difficulties associated with measuring intangible socio-political and cultural drivers of vulnerability, which, if unaccounted for, can lead to an inadequate or even obfuscated understanding of root causes [32, 55]. Therefore, in order to in order to place an emphasis on dynamism and cross-component interactions, while also retaining a focus on socioeconomic, cultural and political conditions, a mixed-methods vulnerability methodology is used (see Methods). The results section is structured around the ways through which multiple stressors combine and compound each other to affect exposure-sensitivity and adaptive capacity over time; numerical values are not applied when attempting to discuss magnitudes of exposure/sensitivity.

### Study area

**Fig 2:**
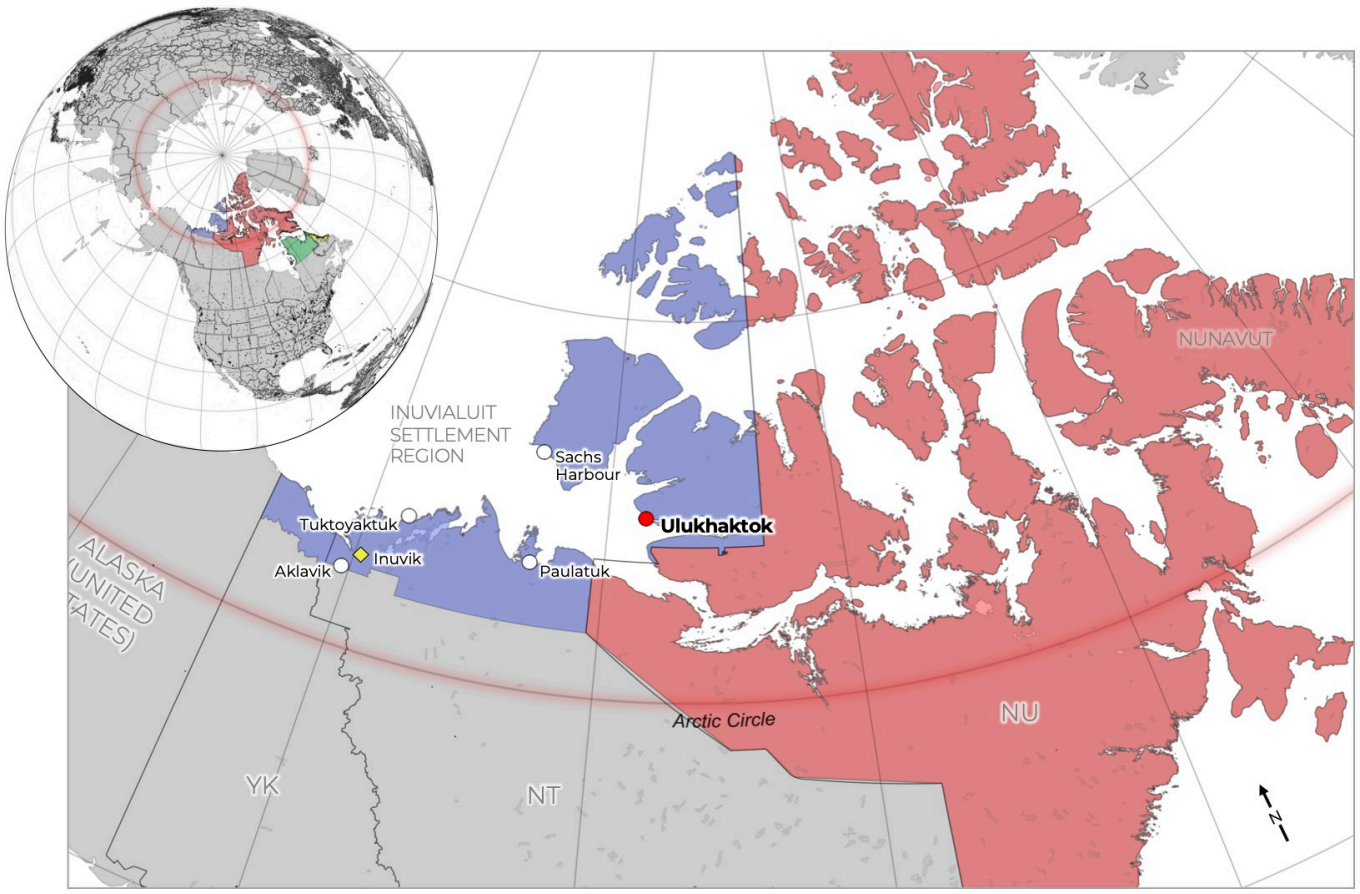
Map of Ulukhaktok and the other five communities within the inuvialuit settlement region of *Inuit Nunangat*. From Naylor et al. [63].

Ulukhaktok is a community of ~440 people located on the western coast of Victoria Island within the Inuvialuit Settlement Region ISR of Inuit Nunangat (western Arctic Canada) [56] (Fig 2). Established as a permanent settlement in the late 1930s with the construction of a Hudson’s Bay Company trading post and a Roman Catholic mission, the construction of buildings at the site initiated a period of drastic livelihoods change and sedentarisation for Inuit living nearby [57, 58]. Prior to the 1930s, the *Kangiryuarmiut* and *Kangiryuatjagmiut* peoples of Victoria Island had practiced semi-nomadic livelihoods, predicated on temporary settlement in seasonal camps and patterns of movement in line with the migration of keystone subsistence species [59, 60]. However, over the following decades a government-subsidised housing initiative in Ulukhaktok, and pull factors related to education and opportunities for wage-based employment, all factored into the sedentarisation of peoples into the community [61, 62].

By 1967, the last family had moved from off the land and into permanent housing [64]. As a result, distal traditional hunting and trapping grounds were abandoned in favour of new or existing areas more proximal to the community, whilst a desire to maintain certain keystone species for economic, cultural and dietary purposes necessitated the uptake of mechanized transport in order to travel greater distances in shorter periods of time [60, 65]. Mechanisation also enmeshed Inuit subsistence practices within the broader global and national economies of resource use and capital accumulation, and by extension, increased the community’s reliance on waged labour, cash liquidity and the global fur market [66–68].

Despite wide-ranging sociocultural and economic changes since the early-to-mid 20^th^ century, and the difficulties now be associated with accessing their traditional lands, many *Ulukhaktokmiut* (peoples from *Ulukhaktok*) still regularly engage in subsistence activities in the present day. The strong ethos and cultural basis that underpins subsistence remains celebrated in the community, and the year-round right to harvest is protected in treaty legislation through the *Inuvialuit Final Agreement*. As of 2018, 75.9% of the adult population in Ulukhaktok stated that they had either ‘hunted or fished’ in the previous calendar year, as compared with 45.4% across all communities within the Beaufort Delta region as a whole [56]. Species, considered integral to the stability of the community foodshed, include muskoxen, *umingmak* (*Ovibos moschatus*), caribou, *tuktu* (*Rangifer tarandus groenlandicus x pearyi* and *Rangifier tarandus*), ringed seal, *nattiq* (*Pusa hispida*), king eider ducks, *qingalik* (*Somateria spectabilis*), Arctic char, *iqalukpik* (*Salvelinus alpinus*), and lake trout, *ihuuq* (*Salvelinus namaycush*). In addition to the by-products of some of the above, polar bears, *nanuq* (*Ursus maritimus*), grizzly bears, *akhak* (*Ursus arctos horribilis*), wolves, *amaruq* (*Canis lupus*), and Arctic fox, *tiriganniaq* (*Vulpes lagopus*), also represent a potential economic resource through the sale of pelts, furs or horn.

Beyond their sociocultural importance, however, subsistence foods are also essential in light of the lack of alternatives available to *Ulukhaktokmiut*. Subsistence foods, typically comprising 50% or more of dietary meat intake [19], are supplemented by store-bought foods in the community, which, while subsidized by the Nutrition North Canada (NNC) program, are often costly, relatively nutrient-poor, and can pose a challenge in terms of affordability [69, 70]. According to the 2019 Community Price Index, prices in Ulukhaktok are, on average, 80% more expensive than in Yellowknife, and 26.3% more expensive than Inuvik, the administrative centre of the of the Beaufort Delta Region [71]. The excess cost of store-bought items in the community stems from the number of food miles they incur; long shelf-life foods are shipped to the community once per year on the NWT Marine Transportation Services (MTS) barge, whilst perishables and fresh foods are flown in each week via Inuvik.

Given the importance of subsistence foods for food security and nutrient intake in the community, changes to the foodshed arising from climate change have become an increasing concern in recent years. Current trends for the Northwest Territories indicate an observed increase in annual mean temperature of 3°C or more between 1948–2016, with mean annual winter warming of between 4–6°C across the same period [72]. Forecasts of future annual mean temperature change across Northern Canada as a whole suggest that further alteration of the environment is inevitable, with projections increases of between 2.1°C to 7.8°C by 2100 relative to 1986–2005 values depending on emissions scenarios [72, 73]. Although previous research in Northern communities has developed baseline understanding of how an altering climate is affecting the vulnerability of food systems [e.g. 74–76], new methodologies focusing on longitudinal methods or real-time community-based monitoring show promise for identifying more nuanced dynamics and interactions between climate and other interacting stressors [35, 77]. The use of longitudinal methods has seen increased application in recent years [e.g. 11, 78], however, the application of real-time approaches, which provide an opportunity for understanding the day-to-day and season-to-season interplay of climatological and broader socially-constructed dynamics remain less widespread.

## Methods

### Data collection

This paper draws upon work conducted as part of the *Tooniktoyok* project jointly developed and led by Inuit in Ulukhaktok in collaboration with researchers. An explicit focus within the project was placed on non-Inuit researchers holding a facilitatory—as opposed to directive—role in the research process, and researcher’s actions were guided by best-practices relating to community collaborative research [79, 80], and guidelines produced by Inuit organizations [81, 82]. Data collection spanned a two-year period between June 2018 –June 2020. A cohort of 10 Inuit hunters, from a range of socio-economic backgrounds within the community and aged 26–82 years, were asked to take part in bi-weekly participatory mapping sessions and semi-structured interviews, conducted as a group and convened by a local project co-ordinator. The cohort were selected through consultation between researchers and the Hamlet of Ulukhaktok based upon purposive criterion sampling [83]. Criteria for selection included hunter experience [84], inferred from the regularity with which participants engaged in land-based activities and their knowledge about the lands surrounding Ulukhaktok, and communication skills. Participants were required to have the time and ability to regularly discuss, in-depth, their experiences of hunting and practicing subsistence. The broad age range of hunters allowed for the incorporation of multiple perspectives beyond those of just elders and facilitated knowledge transfer and co-learning between participants and researchers in order to create a co-produced research agenda.

Each bi-weekly interview and mapping session commenced in an intentionally conversational format, with hunters asked to recount the stories of their hunting trips from the previous two weeks to the wider group. The interview protocol was outlined sequentially according to 5 stages documented in Table 1.

**Table 1:** Semi-structured interview protocol using conversational format for elicitation.

**Fig 3:**
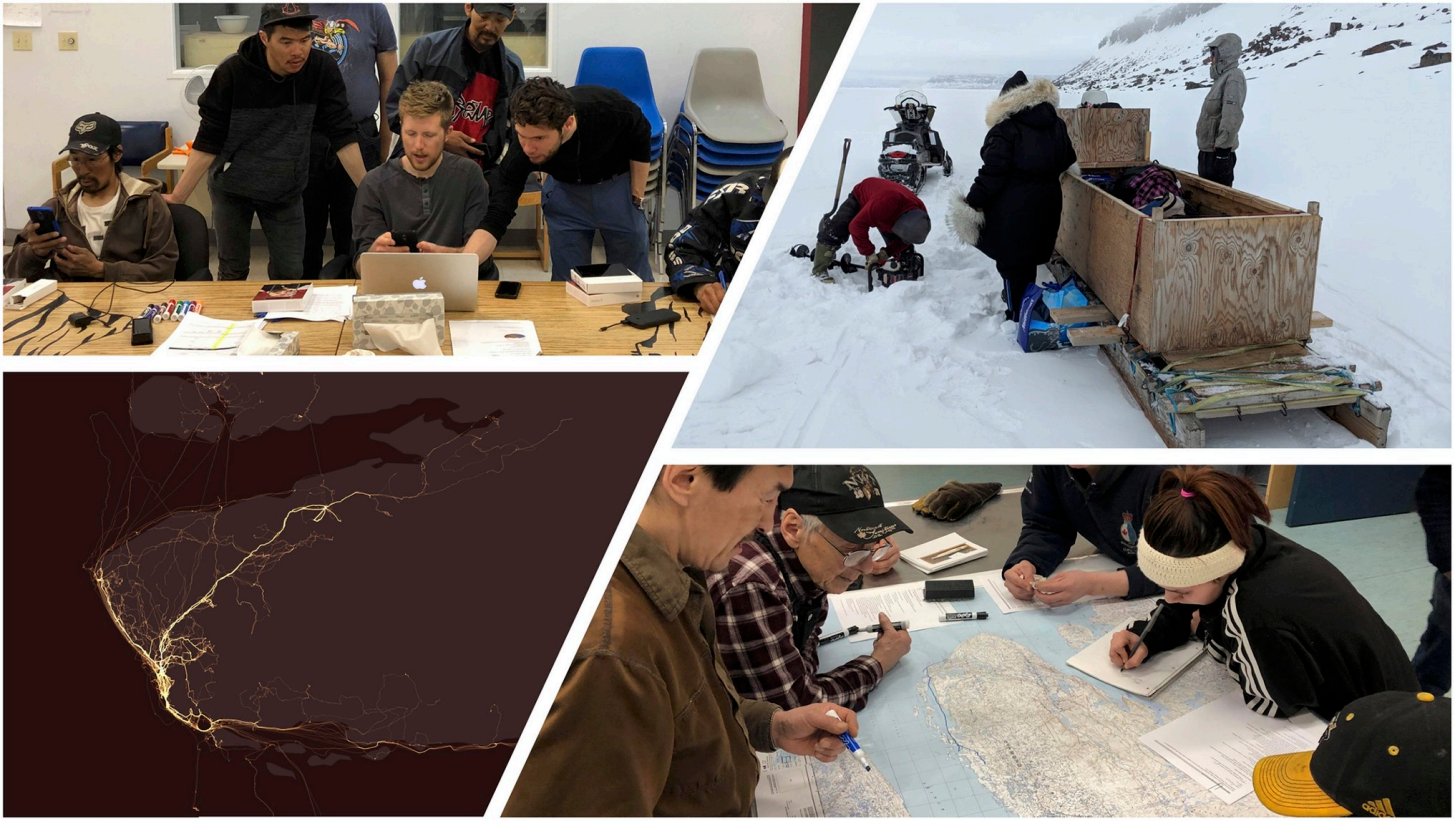
Types of elicitation and data analysis. a): real-time GPS tracking, import of data into QGIS 3.12 and ArcGIS 10.08, b) participant observation, c) participatory mapping and bi-weekly semi-structured/conversational interview. Basemap: *Dark Grey Canvas* (Attribution: Esri, HERE, Garmin, INCREMENT P, © OpenStreetMap contributors, and the GIS user community).

**Stage 1**
Commence session with high level discussion of group activities for the week—each participant notes whether they travelled on the land that week and takes turns tracing and annotating their routes on a set of laminated 1:250,000-scale maps using whiteboard markers (Fig 3).
**Stage 2**
Following annotation, each individual tells the story of their hunts to the rest of the group: recounting in their own words their experience of travelling on the land that week. This offers a chance for participants to highlight observations or feelings to the group that would not otherwise be covered by the more semi-structured nature of follow-up questions, or may not explicitly be addressed by an interviewer with a Western valence. Interviewer makes notes on topics not ordinarily covered by the interview guide where a subsequent line of questioning would add context or valuable information.
**Stage 3**
Interviews develop into a more structured format, conducted between the interviewer and individual participants in the presence of the whole group. Prompts are organised thematically according to the conceptual framework (Fig 1) and according to the notes made on discussions in previous stage. Routes of enquiry centre around the productivity of specific hunting trips (mass of edible weight returned); socioeconomic and other non-biophysical barriers to hunting success or access to hunting grounds; coping mechanisms and potential adaptive measures that have facilitated subsistence activities; and broader scale observations of change that have occurred in the community post-sedentarisation.
**Stage 4**
Interview returns to a more discursive format involving all participants. Cohort are encouraged to ask any questions they might have about each other’s trips or the on any answerers provided in Stage 3. They are also encouraged to offer anecdotes of similar experiences they might have had, with the objective of further developing narratives and contextualising earlier discussions.
**Stage 5**
Interview session concludes with photographing of annotated routes, which can then later be digitised and combined with GPS tracking data using QGIS3.12 and ArcGIS 10.08.

In rare instances where hunters felt less comfortable discussing their routes or experiences in a group setting, one-on-one sessions between members of the cohort and the project-coordinator were offered. All interviews were audio recorded, with the co-ordinator also taking written notes to assist in data analysis. To prevent the privileging of climate as a factor in exposure-sensitivity, and consistent with other work in this area [e.g. 85–87], the phrase ‘climate change’ was omitted from all questioning, and the theme of environmental change was only included in the prompt “did you experience anything unusual on the land whilst you were out”. In line with the collaborative, community-led approach adopted by *Tooniktoyok*, the results section includes verbatim quotes so as to retain the narrative and granularity of participants’ stories (further supporting quotes are also available in S1 Table).

Conversational interview formats in the manner outlined above, which have a purposeful direction and topic but can also digress or ‘meander’, are sometimes referred to as ‘research topic yarning’ in other Indigenous-focused research methods literatures [88, 89]. Conversational methods are a legitimate and culturally appropriate means of interviewing Inuit participants, aligning with oral traditions surrounding knowledge transmission, and allow for Inuit values and perspectives to be conveyed into the research process, creating relationality and accountability between interviewee and researcher [90, 91]. Due to COVID-19, interviews and participatory mapping sessions were stopped April 2020, with data collected during this period through the remaining monitoring approaches outlined below only.

In addition to participatory mapping sessions and semi-structured interviews, the hunter cohort were also asked to track their harvesting activities across the project through the use of a GPS tracking system between June 2018-June 2020 (Fig 3). The GPS system was internet-linked, for use with low bandwidth, with hunters asked to upload their recorded routes as.gpx files to a secure cloud system, which both researchers and the cohort also had access to. Hunters were given full control over the data they uploaded and were given the option of either hiding their routes from other hunters within the system or not having their routes included in the creation of final maps. For participants who did not have access to the internet at home, the Helen Kalvak School Wi-Fi system was made available for the uploading of data. From tracked trails, meta-data relating to distance travelled, seasonality, time and date, and location of harvests were extracted.

Potential ethical issues relating to the provision of GPS systems (including possible increases in risk-taking behaviour [92, 93] were reduced through the use of a passive system, which could track activities but could not be used for navigation. With regard to critiques over the potentially ‘undemocratic’ nature of past GIS research [94, 95], results and associated maps were regularly shared with participants. In relation to perceived incompatibilities between GIS and Indigenous value systems (see [96, 97]), the mixed-methods nature of this research meant that trails tracked using GPS were also hand-drawn by the cohort in participatory mapping sessions; therefore, a process of ‘interviewing the map’ was undertaken (see [98, 99]). Combining routes with an oral recounting of hunting trips allowed for trail data to be contextualised and facilitated the conveyance of Indigenous values and worldviews about the traversed environment that would not ordinarily be captured through conventional, more technocentric participatory GIS studies [97, 100].

In addition to the above, more implicit methods of research were also adopted in order to triangulate data collection and analysis. These focused on participant observation, and included ‘deep hanging out’, whereby members of the research team—who spent a total of approximately 12 months in the community between June 2018-January 2020—lived with families in Ulukhaktok and took part in hunting trips. Participant observation through immersion represents a critical means of dispelling preconceptions or *a priori* assumptions that may have arisen from receiving second-hand information before the conduct of research and was important for developing routes of inquiry in semi-structured interviews and discussions that were germane to the research question at hand [101, 102].

Primary research data was supplemented by secondary weather and sea ice data relating to indicators of environmental change. These were extracted from two weather stations that had been established at the community airport, one running from 1987–2009, the other from 2010-present, in addition to daily ice charts published by the Canadian Ice Service (CIS). Dates for sea ice break-up and freeze-up respectively were established according to the average day of the year on which when concentrations at eight points in Amundsen Gulf and Prince Albert Sound dipped below 5/10 (50% surface cover) in the summer months, and above 5/10 in the winter months (see [11, 103]).

### Data analysis

GPS data were imported into QGIS and ArcGIS software to identify distances travelled and hunters’ annual and seasonal land use patterns. Place names, the location and mass of harvests, and other features of interest that were annotated during mapping sessions relating to land conditions or events occurring on hunting trips were also added. GIS maps were shared on a quarterly basis with the cohort, and prior to project conclusion were printed out to allow for direct annotation of changes and amendments to place name spellings. Upon finalisation, routes and hunting areas were compared with historical maps land use activity in the region (see [104]), and copies were provided to major community organisations (Hamlet of Ulukhaktok, Ulukhaktok Community Corporation, Helen Kalvak Elementary School).

Interview data were transcribed by researchers involved in producing interview formats and interviewing participants. Once complete, transcripts along with research diary entries were uploaded to NVivo 12 and analysed through latent content analysis. Content analysis took the form of Provisional and Structural Coding, whereby a provisional list of themes identified as drivers underlying adaptative capacity, adaptation, and exposure-sensitivity, derived from this study’s conceptual framework (Fig 1) and previous literatures on Arctic climate vulnerability research, were coded and indexed according to the driver in question, its influence on components of vulnerability, and its relative dynamism (e.g. “adaptation—slow: Indigenous knowledge relating to food preparation” or “exposure sensitivity—fast: environmental change relating to land access”). Particular emphasis was placed on situating narratives discussed in interviews within the broader context of data derived from weather stations, participant observation, and previous research on environmental change in the community [e.g. 13, 67, 78]. Weather and climate data were graphed and, where granularity was sufficient, were subject to statistical analysis through least squares regression and the Mann-Kendall test, with *p* = <0.05 as the threshold of significance. Interview data were anonymised; in instances where names are used included quotes, this has been altered to a pseudonym. Due to the impact of COVID-19 on international travel in 2020, results were provisionally communicated to participants digitally and through the local project coordinator.

### Ethics

Research was undertaken with consent of the Hamlet of Ulukhaktok and was overseen by a four-person volunteer Inuit Oversight Committee within the community. Study protocols were approved by Institutional review boards at the University of Guelph (REB 17-12-012) and the University of Leeds (AREA 18–117). The research was licensed by the Aurora Research Institute (No 16533), which oversees research in the Northwest Territories. Verbal and written consent for interviews and data storage was obtained from each participant.

## Results

### Altering subsistence land use patterns in Ulukhaktok

**Fig 4:**
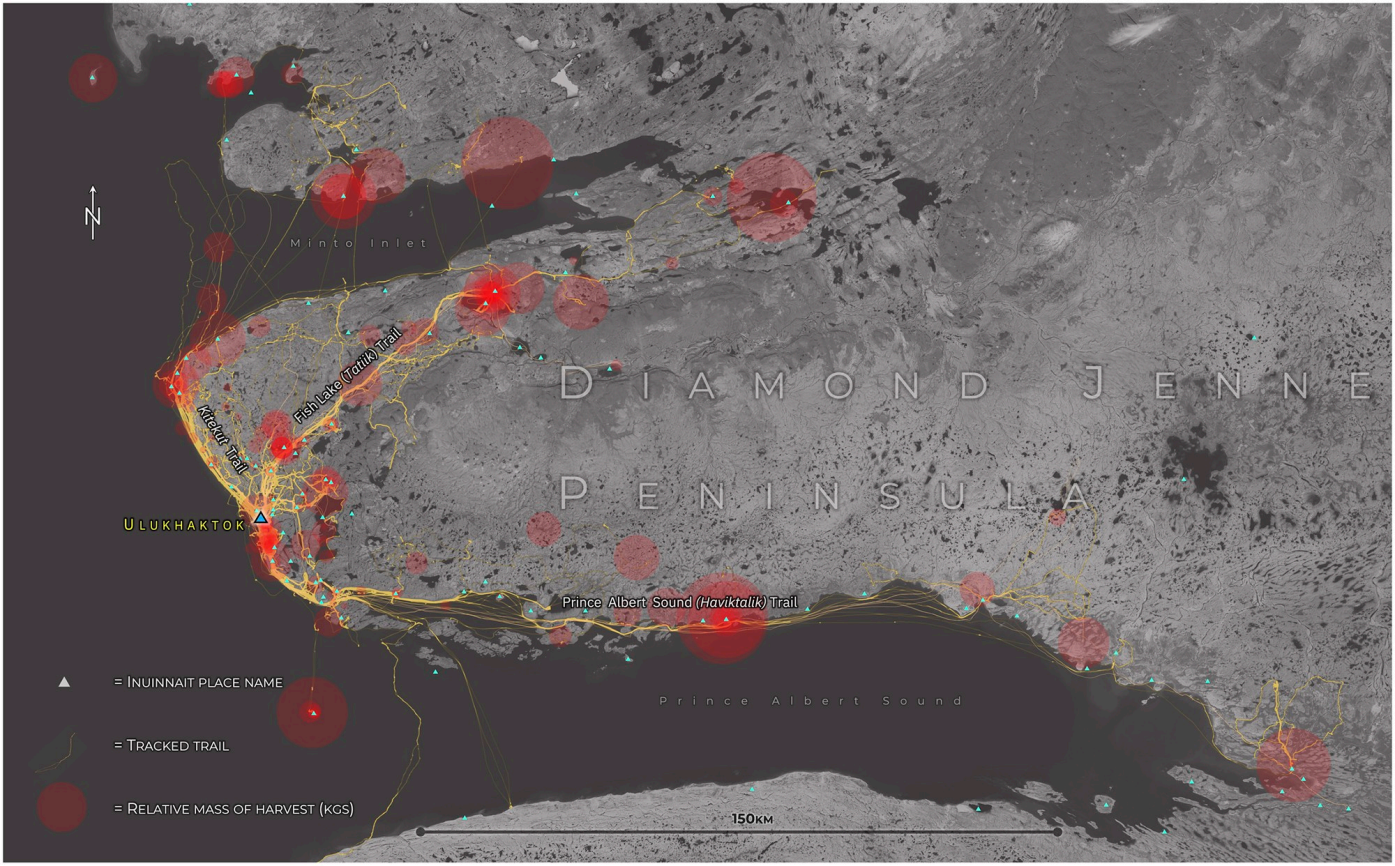
Annotated and tracked GPS trails around Ulukhaktok June 2018—June 2020. Includes locations of Inuinnait places visited and harvests and their relative mass of edible weight (kgs), recorded for the year 2019. Basemap: Esri World Imagery (Attribution: Esri, Maxar, GeoEye, Earthstar Geographics, CNES/Airbus DS, USDA, USGS, AeroGRID, IGN, and the GIS User Community).

Between 1^st^ June 2018 and 1^st^ June 2020, 409 routes were recorded by the cohort, yielding an average of 0.56 routes per day of data collection. 376 of these were recorded directly through GPS monitoring—from which exact distance measurements could be extracted—and a further 33 routes were annotated onto maps during semi-structured interviews. Due to the large scale of the maps used for annotation (1:250,000), and due to some hunters opting to provide only summary sketches of their travels, exact distance measurements were not extracted from drawn routes. In total, 23,995.58km of routes were recorded in the GPS system (Fig 4), with snowmachines covering the greatest distance (12, 819.17km), followed by boats (6,248.89km), and ATVs (4,927.52km). Snowmachines were also used with the greatest frequency (in 215 (53%) of the 409 instances for which mode of transport was recorded by hunters). ATVs (*n* = 111 / 27%) were used more often than boats (*n* = 83 / 20%), with the latter involving, on average, travel over the greatest distances (Table 2).

**Table 2:** Distances travelled by year, mode of transport as part of the *Tooniktoyok* project, June 2018-June 2020.

	GPS Trails (additional annotated)	Distance Travelled (kilometres)^c^ (% of total distance)				Date of Break-up^d^	Date of Freeze-Up^d^
		ATV	Boat	Snow-machine	Total		
**Year**							
2018	178 (0)	3,528.11 (40%)	3,741.15 (42%)	1,617.17 (18%)	8,886.43	June 30^th^	Oct. 14^th^
2019^a^	162 (33)	1,399.41 (11%)	2,507.74 (19%)	9,054.98 (70%)	12,962.13	June 19^th^	Nov. 7^th^
2020	36 (0)	0 (0%)	0 (0%)	2,147.02 (100%)	2,147.02	-	-
**2018–2020**	**376 (33)**	**4,927.52 (21%)**	**6,248.89 (26%)**	**12,819.17 (53%)**	**23,995.58**	**-**	**-**
**Post-break-up (Summer)**							
June 30^th^ 2018—Oct. 13^th^ 2018	131 (0)	3,150.19 (45%)	3,741.15 (54%)	46.96 (1%)	6,938.3	June 30^th^	Oct 14^th^
June 19^th^ 2019—Nov. 6^th^ 2019	43 (14)	1,251.94 (29%)	2,384.54 (56%)	660.56 (15%)	4,297.04	June 19^th^	Nov. 7^th^
**Summers 2018, 2019**	**174 (14)**	**4,402.13 (39%)**	**6,125.69 (55%)**	**707.52 (6%)**	**11,235.34**	**-**	**-**
**Pre-break-up (Winter)**							
31^st^ Oct. 2017—June 29^th^ 2018^b^	4 (0)	131.65 (94%)	0	8.62 (6%	140.27	June 30^th^ 2018	31^st^ Oct 2017
Oct. 14^th^ 2018—June 18^th^ 2019	136 (17)	393.74 (5%)	123.2 (1%)	7,820.11 (94%)	8,337.05	June 19^th^ 2019	Oct. 14^th^ 2019
Nov. 7^th^ 2019 –^b^	62(2)			4,282.92 (100%)	4,282.92	-	Nov. 7^th^ 2019
**Winters 2018/2019, 2019/2020**	**202 (19)**	**525.39**	**123.2**	**12,111.65**	**12,760.24**	**-**	**-**

^a^ Denotes a full calendar year, as opposed to 2018, 2019, which denote June 1^st^—Dec. 31^st^.

^b^ Data collection period does not span entire ‘winter’ period.

^c^ Denotes GPS-tracked routes.

^d^ For process of deriving freeze-up and break-up dates, see Methodology.

The cohort’s recorded and annotated trails covered a calculated land use area of approximately 27,940.857 km^2^ (including land, sea and ice) between 2018–2020, and the maximum extent of distance travelled away from the community (annotated) was 250.22km as the crow flies, with the maximum recorded total trail distance recorded by GPS being 525.26km. These statistics provide a strong proxy measure of utilised contemporary foodshed extent. When compared with historical maps produced by Freeman [104] and Collignon [60] that examine hunting range on the lands surrounding Ulukhaktok for the harvesting periods ‘1930’s-1965’ (Period I (see Collignon [60]) and ‘1965-late 1970s’ (Period II (see Collignon [60]), data on areal extent is indicative of a decrease of approximately 83,301km^2^ (74.8%) and >26,733km^2^ (48.9%) respectively. Also of note was an observed reduction in the relative diversity of routes used by the cohort compared with these periods (trails from historical periods inferred by location of traplines) [104]. Between 2018–2020, the majority of travel for harvesting activity was confined to three main trails. The Prince Albert Sound trail (Aug-Oct and Dec-Feb caribou and muskox hunting, Nov-March and Jun-Aug sealing) to the southeast; to the northeast, around and on the trail to Fish Lake (*Tatiik*) (Sept-Jul muskox hunting, Jul-Aug and Oct-Dec char runs, year-round lake fishing); and to the northwest on the coastal trail toward *Kitekut* (May-Jul duck and fowl hunting, Nov-March and Jun-Aug sealing) (Fig 4). (Dates represent approximate periods of hunting activity, for full seasonal calendar see Parker [105].)

### Current exposure-sensitivities and adaptive capacities affecting the foodshed

#### Climatic variability affecting land access

In addition to a constriction of the foodshed over a longer timescale, a reduction in the areal extent of harvesting at an inter-annual scale was also evident within the study period itself, during the winter of 2018/19, when delayed and limited snowfall to the east of the community left rocky ground exposed or insufficiently covered. The cohort noted that this affected their ability to harvest caribou through creating land-use bottlenecks, with a number of traditional trail routes compromised and detours were required, and also had implications for travel safety and the accumulative costs associated with the replacement of skirods, track wheels, and hyfax runners. Hunter observations of reduced snowfall broadly reconciled with descriptive historical weather data. In any given winter since 2004/05, the recorded ground snow thickness between 1^st^ Oct– 1^st^ May in 2018/19 was lower than in 12 of previous the 14 years, was the lowest on record since the 2010/11 season (11.15cm (*n* = 213) vs. 9.18cm (*n* = 181)), and was 39.8% lower than the average daily mean across all days between 1^st^ Oct– 1^st^ May between 2004–2018 (S1 Fig).

“It’s good all the way up to here [down Prince Albert Sound], but then once you get on the land [there] was no snow. The [sea] ice was okay, but it doesn’t take long to finish skirods over here.”

(20^th^ June, 2019; #441–07)

Observations pertaining to changing snow conditions in winter of 2018/19 were accompanied by perceptions that wind and ice and land conditions were becoming less predictable in general, reducing the number of days available for travel and with implications for the applicability of previously established ecological knowledge. Participants noted that the shoulder portions of seasons were particularly severe, with freeze-up commonly cited as taking longer or, in the case of winter 2018/19, incompletely as a result of changing wind conditions, requiring some to take greater risks to harvest. Alternatively, there were perceptions that breakup could now occur rapidly due to increased wave strengths, but that in some cases winds would keep broken-up ice in bays and inlets, meaning that neither snowmachines nor boats could be used for travel. Changing melt season dynamics were also observed on the land, affecting permafrost dynamics and the amount of standing water and overflow, with implications for ATV and snowmachine trail access.

“The biggest thing for the ice this year is how long it’s taking to freeze up…. The ocean is taking so long to freeze, and the ice isn’t staying when it does freeze. It opens up [again]. It’s been opening up all winter…ice is not getting thick enough anymore I guess… It was like this last year too, but it didn’t last as long; this time it’s lasting longer.”

(20^th^ March, 2019; #117–06).

**Fig 5:**
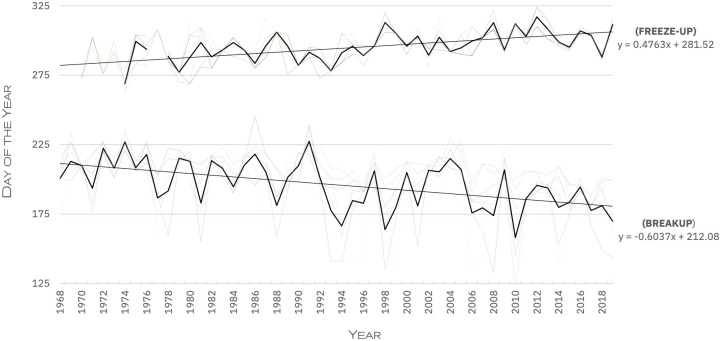
Day of the year on which break-up/freezeup occurred on the waters around Ulukhaktok, 1968–2019 (adapted from Fawcett *et al*. [78]).

Hunter observations were supported to a degree by available sea ice data, which indicates a trend toward later year-on-year ice freeze up (Fig 5). However, Mann-Kendall analysis of de-seasonalised max and mean annual temperatures and between 1987–2010 and 2000–2010 from two weather stations in the area, and an analysis of maximum wind speed between 2003–2020 indicates no trends with statistical significance (*p* = <0.05). It is possible that average wind speed and direction has changed significantly in the community, however, limited data availability from either weather station precludes these from analysis.

In terms of adaptations, much of the cohort cited the use of alternate travel routes or travelling at a different time as primary means of overcoming land use access problems. Others suggested that they had purchased, or in the future intended to purchase, larger boats with four-stroke engines. Larger hulls permit travel in winder conditions, while four-stroke engines are typically more fuel efficient, thereby offsetting the increased costs associated with traveling through larger waves and swells. Notably, almost all of the adaptations cited for responding to changing climatic conditions required additional economic input (i.e. the purchase of new machinery, or the use of more supplies).

#### Mechanical issues, vehicle design, supplies used

Mechanical reliability was recurrently noted as a factor affecting the day-to-day accessibility of the foodshed. In the 2019 calendar year, of 130 trips (out of a total of 132) where the question of reliability was raised, participants stated that they had experienced mechanical issues relating to either their snowmachines, ATVs, boats, sleds or other hunting equipment 18.4% of the time. This was notably higher than the incidence of issues experienced pertaining to the environment, which were recorded in 13.6% of cases across the same period. Mechanical issues rarely resulted in a trip being cancelled or ended, as often hunters were able fix machines, or instead travel on the sleds or vehicles of those accompanying them. The majority of issues pertained to the breaking of snowmachine ski rods or hyfax runners due to insufficient snowfall in winter of 2018/19 (as above). However, seven instances of blown pistons in machines were noted, and in three cases hunters were forced to attempt to return to the community by foot, having left their machines on the land. In summer of 2020, for instance, a member the cohort was forced to walk approximately 25 kilometres back to the community, in rain and fading light, following the breakdown of their companion’s ATV and due to their own vehicle becoming stuck in boggy ground.

Compounding the increased risk of damage to machines in poorer conditions, hunters remarked at the prohibitive cost of new parts and supplies (e.g. gasoline, heating fuel) as key factors affecting land access. From an intra-community perspective, differential sensitivity relating to the availability of gasoline, parts and back-up machinery was especially notable between different sharing networks. Members of the cohort with access to the most capital-rich networks often demonstrated the greatest adaptability, either through their ownership of, or access to, multiple modes of transport, or through an ability to borrow parts or gasoline from other members of their family in order to access the land when they themselves had run out. Conversely, a lack of parts, insufficient funds to purchase gasoline or other supplies, or the fact that the equipment needed to be shared across multiple members of a network simultaneously, limited access to the foodshed among members of the cohort with less financially robust networks—sometimes impacting harvesting for days at a time. Some participants felt an inability to access land in this manner held implications intergenerational knowledge transfer, with subsequent cascading effects for availability the of subsistence species (i.e. knowledge of how and where to hunt).

“There’s a lot of things we’ve got to teach our kids, and not all of us are going to teach them because some of us hasn’t got the equipment to take our families out.”

(7^th^ July 2018; #008–01).

A notable adaptation to offset the excess cost of gasoline among high-capital members of the cohort has been investment in more-fuel efficient four-stroke snowmachines, ATVs, and boats. Although there was also a perception that these new vehicles, particularly snowmachines, were less resilient to the changing environmental conditions due to the design of new machines catering more to sports and leisure markets. Spikes in exposure-sensitivity, derived from difficulties sourcing or paying for parts or the obstacles related to the operation or fixing machines on the land, often necessitated ‘bricolage’ adaptations—making do with what resources are at hand—in order to complete trips or access the land (see [106]). Over time this has resulted in adaptive learning and increased preparedness, whereby hunters frequently take a ‘toolkit’ of various items, such as rope, tyre plugs, duct tape, spark plugs, etc. that could often be used to conduct interim repairs. Examples of bricolage adaptation included one participant wrapping a towel around the handlebars of his machine when electrical wires short-circuited and heated the metal in them, the use of ropes to support broken vehicle suspension chassis, piling sea ice (*hiku*) on top of engine blocks in order to cool them when antifreeze did not work, and the creation of new machine parts (such as skirods) from scrap metal. Maladaptations were evident in some cases, when members of the cohort used bricolage adaptations beyond their optimal lifespan, resulting in more severe damage once poor conditions were again once again encountered. In addition to bricolage toolkits, participants often discussed that they had taken additional supplies with them onto the land, or had stored supplies (e.g. gasoline, heating fuel) at caches. Increased preparedness in some cases also allowed for opportunism when harvesting, often allowing hunters to catch more than one species when travelling.

“I tried to come back [to town] a couple of times when we were out. First time, past *Nuvuk*, the wind kept picking up…really started getting rough so we had to turn back. The repairs we did this summer [to the boat], they got wet… they were short repairs [in the hull], but [they’re] really long cracks now. Really lots of water going in [to the boat].”

(27^th^ September 2018; #033–10).

#### Inter-annual variations in species availability and quality, multiple exposures

Hunters perceived that they were seeing an increase in the number of parasites or disease that they were seeing within certain species, particularly within muskoxen and char populations. In many cases this was attributed to environmental changes, and in some cases explicitly to climate change. An observed increase in the presence of parasites is consistent with recent brucellosis and lungworm research conducted recently on muskox populations across Victoria Island [107]. Increased incidences of disease hold implications both for species availability, as some parasites can leave animals more susceptible to predation, but also from a food quality perspective; brucella-infected meat can be particularly dangerous if consumed, for instance [108, 109].

Observed changes in muskox and health were also accompanied by suggestions that populations may be in decline, or less available through the migration of populations toward the more distal areas of the foodshed. Despite a relative constriction in foodshed extent since the 1970s and 1990s (see ‘Altering subsistence land use patterns in Ulukhaktok’), a number of hunters suggested that they were travelling further in attempts to harvest muskox. Some hunters attributed decline in muskoxen availability to a return of limited caribou populations to areas more proximal to the community and suggested that the two populations were on an inverse cycle. These observations reconcile with research conducted by Fawcett *et al*. [78], who in 2016 documented perspectives that muskox populations were found further from the community, and that, as a result, that some hunters expected to subsequently see a rebound in the Peary caribou population (see also [75, 110]).

“We have to go quite a ways… for what we’re trying to get. The other day, like I said, me and my daughter went out to go and hunt muskox, and we went over 100 miles return [journey], and we haven’t seen any muskox… this place is already hunted out.”

(31^st^ July 2019; #505–06).

“It’s getting harder… but the caribou are coming back, so that’s making up for the muskox disappearing…”

(7^th^ March, 2019; #163–11).

In 2016 the community was assessing the feasibility of expanding the number of Arctic char that could be commercially caught, with a proposed increase from 500 to 700 fish allocated for sale per annum [78]. Char numbers remained relatively high through 2017, whereafter in 2018 the community’s Char Working Group made a decision to increase the commercial quota to 700 for the following year. However, a number of the cohort throughout the study period (2018–2020) perceived a decline or highlighted unpredictability with the fluctuation in char numbers—the root cause of which is not as yet fully known. As a result, the Char Working Group returned the tagged quota to 500 for the 2020 season, and subsequently placed a moratorium on commercial char fishing between 2020–2025.

Concerns over depressed char numbers were compounded in early-mid July of 2019, when the char run—during which char migrate from the ocean into lakes to spawn—coincided with an anomalous bloom of pelagic tunicates extending approximately 225km along the western coastline of Diamond Jenness Peninsula [111]. A type of small jellyfish, whose presence in the 2019 was attributed to altered ocean currents as a result of early sea-ice breakup, the effect of pelagic tunicates on the physiology of marine animals is poorly understood [111]. A number of the cohort in Ulukhaktok voiced concerns that fish and marine mammals may be avoiding the tunicates (and therefore the coastal fishing grounds proximal to the community), while others suggested that the accumulation of algae on nets was making them more visible to fish, and that this was affecting the viability of fishing. Not only did nets need to be cleaned more regularly—achieved by placing them on the beach and rubbing them with sand—they were also more difficult to physically pull from the ocean due to the added weight of the tunicates; a factor some participants felt was further exacerbated by increased strength of waves in recent years due to climate change (see [112]). The bloom in Amundsen Gulf is reflective of a similar trend of rapid biological change recorded in the Pacific Arctic between 2017–2019, instigated as a result of altered water column temperatures.

“There’s just so much [(tunicates)], all together. I think when animals dive in it goes on their face, that’s why there’s not much seals in the water. I brought the pilots to the airport, and they told me this [there are] thousands of seals on the ice, down Minto [Inlet]. My wife and I went down here yesterday [to the southeast]; lots of seals on the ice. They don’t want to go in the water. Along the shore, in the shallow spots it’s okay. You go a bit further out, there’s millions of that stuff [(tunicate blooms)].”

(4^th^ July 2019; #448–12).

#### Cultural change, broader impacts of globalization

Engagement in the wage-based economy—which has now almost become a prerequisite for purchasing adequate hunting equipment—and its associated time commitments were considered to compound less predictable weather conditions and the greater travel distances required to harvest some keystone species. In some cases, the effect of the wage-based economy was direct, whereby hunters could not travel on the land because they were working. For hunters that were part of either single-parent households, or households with children where the spouse was employed, childcare commitments were also a limiting factor. More experienced hunters within the cohort remarked that less time available for land-based activities was leading to some younger harvesters travelling in conditions that were considered dangerous, at unusual times, or without sufficient preparation. Reinforcing the notion of limited time available for hunting, single-day hunting trips accounted for 64.4% of trips (*n* = 85 / 132) for which interviews were conducted among the cohort in the 2019 calendar year. This may also explain to an extent the recorded constriction of the foodshed in terms of distances travelled from the community relative to studies from the 1990’s and 1970s and has significant implications in light of recent unpublished research establishing association between the number of days hunters spend on the land and the productivity of their hunting groups in the community.

“We were going to go up here and look for eggs too today, just near Fish Lake [(*Tatiik*)]. But we didn’t get to go anywhere… my partner was working [and the kids were at home from school because of the summer break].”

(11^th^ June 2019; #393–05).

Cultural change, in terms of the ways through which youth engaged with the subsistence economy, was voiced as a concern by a minority of the cohort when they were asked about the future of the food system. Opinions occasionally reflected past research conducted by Ford *et al*. [86], among others [87, 113], across Inuit Nunangat whereby youth were perceived to be losing interest in harvesting, or now have diets that primarily predicated on store-bought foods, with implications for knowledge transfer relating to food preparation. However, in many cases the cohort often reflected positively on youth engagement within the *Ulukhaktokmiut* foodshed. This may point to the success of more recent attempts to engage youth in subsistence practices across the Inuvialuit Settlement Region and the wider Arctic, which mainly come in the form of education programmes and increased on-the-land learning [114]. In testament to the continued need for such initiatives in the region, participants suggested that rather than cultural change or a lack of desire to learn, the greatest barrier to youth engagement was the availability of equipment and supplies, or the ability of parents or mentors to take them out on the land (see also [115]).

#### Institutional drivers

Hunters remarked on the fact that institutional support for hunting could be a critical factor in facilitating or constraining land access. In particular, federally funded, community-based initiatives, such as the Community Harvesters’ Assistance Program were perceived positively; in part due to the generosity of grants, and a belief that resources were allocated with the greatest impartiality under such schemes. Conversely, concerns were more frequently raised as to the efficacy of federally and regionally administered programmes, particularly NNC and the Inuvialuit Harvesters’ Assistance Program. Regarding NNC, hunters echoed concerns flagged in other Northern communities in suggesting that the focus of the Canadian Government on funding the provision and affordability of store-bought foods was diverting funds that could otherwise be spent funding harvesters directly, or through developing land-based learning initiatives [69, 116, 117].

“IHAP [(Inuvialuit Harvesters’ Assistance Program)], it’s supposed to be for harvesters… There’s people who get equipment but then don’t hunt, they just use it around town…. the way I see it is that it should be for people that [actually] go for harvesting, not just around town… it should be more looked into for future generations. It needs to be fixed and dealt with.”

(30^th^ June 2019; #504–07).

“CHAP [(Community Harvesters’ Assistance Program)] funding is the one that helps out people. Each household gets at least a barrel (45 gallons) of gas. It usually goes about four times [a year] I think—Spring, Summer, Winter and Fall.”

(31^st^ July 2019; #503–06).

In addition to federally and regionally administered programs, the community also funded a number of on-the-land learning initiatives for youth through the Helen Kalvak Elementary School and the Ulukhaktok Community Corporation (UCC). These projects were seen as hugely successful by the hunters, and often provided opportunities for temporary employment, access to funds, and the creation of social networks that facilitated hunting and land access. In one instance, a hunter who worked closely with the school was also able to borrow one of their snowmachines in order to go trapping whilst his was being repaired. In another, the UCC purchased supplies (gasoline, food) for hunters to take youth on a four-day muskox hunt, with youth allowed to keep the meat and hides they harvested.

## Discussion

This article documents and examines the spatiotemporal vulnerability of a complex adaptive system to climate change in the context of multiple interacting stressors. While complimenting a body of pre-existing scholarship relating to climate change impacts, adaptation and vulnerability in Arctic Canada, it is both conceptually and methodologically distinct. The spatial component of our research provides empirical evidence for the areal constriction of the *Ulukhaktokmiut* foodshed relative to its past extent documented in the mid-to-late 20^th^ century. GPS tracking shows that hunters are travelling less far as compared with early periods of settlement (1930s-1965/1965-late 1970s) and indicates an overall decrease in the diversity of travel routes taken. Although changes in land use have been discussed in previous qualitative studies, this has not been previously quantified. Comparisons of harvest data from 1989 and 2019, population growth of 386% in the community between 1963–2019 [56, 118], and the high rate of recorded hunting participation in Ulukhaktok relative to other Beaufort Delta communities (75.9% vs. an average of 45.4%), suggest that a reduction in harvesting range is not attributable to an overall reduction in the frequency or volume of harvesting by the community. Rather, these trends are indicative of contemporary hunting activities occurring with a similar if not greater intensity, but across a smaller, concentrated area. This is supported by previous research, which states that sedentarisation in the community had the effect of reducing the number of seasonal camps and resupply points in distal locations away from Ulukhaktok [60, 62], and brought about a number of sociocultural changes (e.g. wage-based employment) that fundamentally altered the nature of harvesting. Indeed, following sedentarisation in the 1960s, whole-system re-organization became necessary in the community in order to allow subsistence practices to reprise their essential role in sustaining Inuit livelihoods and food sovereignty within the foodshed [65].

Multiple points of emergent change stemming from sedentarisation and the associated adaptive responses and new exposure-sensitivities that developed across multiple dimensions of the foodshed as a result are still extant and in evidence in many of our findings. Socioeconomic stressors relating to wage-based employment are but one pertinent example—representing a forced adaptation that funds the cost of contemporary hunting technologies and supplies—while also holding the maladaptive effect of limiting time on the land. The real-time monitoring nature of our work allowed us to draw specific conclusions relating to the frequency with which economic factors in particular are affecting the foodshed. Beyond the issue of simply purchasing gasoline to travel, in 2019 hunters recorded issues relating to mechanical problems 18.4% of the time when travelling on the land (26% more often than environmental issues were experienced), many of which necessitated the purchasing of replacement parts. This illustrates the financial capital individuals often require in order to adapt and the close links that now exist between harvesting and exogenous capital markets, and the subsequent potential for differential vulnerability between hunters, particularly between those engaged in wage-based labour and those hunting full-time. The prominence of economic stressors adds to a body of previous research conducted in the community that has pointed to pre-existing tensions between the subsistence economy, wage-based labour and Westernisation [19, 78, 110].

In the context of a changing climate, reduced time on the land stemming from engagement in the wage-based economy, the costs of hunting, and Westernisation—compounded by a reduction in both areal extent and diversity of trails used—makes an understanding of the ways through which the biophysical environment may be changing and affecting harvesting all the more important. Particularly as a reduction in the number of trails or overall hunting range used by hunters also holds implications for the diversity of areas that hunters are able to use and wild species that can be accessed. Such diversity and redundancy potential has been identified as a key factor historically underpinning adaptive capacity across Arctic communities, and alterations to land use intensity in tandem with climatic changes holds implications for placing subsistence species under strain from multiple stressors [119]. To this end, our study documented two types of climatic drivers that could act as landscape and ecosystem stressors: high-magnitude low-frequency events or more incremental, accretionary year-to-year changes. Previous research in Northern Canada has explored the notion of multiple stressors on food systems in the context of anomalous climatic extremes (e.g. [76, 120]) and broader incremental changes (e.g. [75, 115, 121]). However, the temporally constrained nature of data collection in past scholarship means that these have infrequently explored within the same study. Among the cohort here there was widespread recognition that changing climatic conditions, both incremental and anomalous, were developing new and unexpected challenges. This was seen to be particularly true for the biophysical extremes, which often produced considerable spikes in exposure-sensitivity, and in some cases exceeded the coping or adaptive capacities operationalised by individuals and social networks. One example was the anomalously low snowfall recorded to the east of the community during the winter season of 2018/19. This resulted in the creation of significant land use bottlenecks and drastically increased wear and tear on expensive machinery, with implications not only for the period over which an adequate land area could be accessed for hunting keystone species, but also the financial viability of harvesting for some families who were simultaneously experiencing compound economic stresses relating to the cash liquidity. The longer-term implications of such ‘spikes’ is poorly understood and represents a priority for future research.

Incremental biophysical changes were most frequently discussed in conjunction with other socially constructed stressors. Indeed, notwithstanding the potential impact of incremental climatic changes as standalone stressors on the foodshed, rather than being the cited as the most prominent or severe drivers of exposure-sensitivity, much discussion of gradual environmental change by the cohort was contextualised by its role in exacerbating pre-existing social drivers of vulnerability and creating cascading effects. Two prominent examples included: *i*) travelling in sub-optimal conditions, and *ii*) the alteration of animal distributions or populations. Regarding the former, travel in poorer conditions, be these adverse wind or weather or deteriorating trails, was most commonly discussed in the conjunction with their effect on fuel efficiency (and subsequently the high fuel costs within the community), or the issue of available time when considering travel on an alternative day. The latter often left hunters with the choice of “risking it” if conditions were poor at weekends, or instead forced travel in evenings after the working day had concluded. The alteration of animal distributions or populations, on the other hand, was often contextualised by political factors relating to wildlife management policies and concerns over the provision of support to hunters by institutions if hunters have to travel further to access certain species. The latter was particularly in relation to the costs associated with equipment and consumables, in addition to the eligibility criteria of individuals for harvester support. These findings, in addition to a lack of statistical association to support some observations, give credence the arguments that stimuli deriving from the effects of climate change are not always the most salient issues affecting Arctic communities on a day-to-day basis [12, 85, 86, 122, 123]. Indeed, discussions relating to exposure-sensitivities often rather than mentioning climate or environmental change as a primary driver, centred around the economy and wider socio-political stressors relating to vulnerability.

In the absence of sufficient individual financial capital to repair machines or purchase fuels and other supplies to access the land, many hunters cited a reliance on either their social networks or institutional support; thereby demonstrating the crucial importance of strong social relationships to the continuing stability and redundancy potential of the foodshed. Many harvesters in Ulukhaktok, in addition to sharing country foods, also shared their equipment between familial groups. Therefore, particularly for younger hunters, the ability of older members of their networks to invest in equipment, or to have spare machines that might otherwise allow for redundancy across a sharing network, has become crucial. In addition to the differential strength on individual hunter’s financial capital affecting their exposure-sensitivity, the relative wealth and strength of a harvester’s social network also therefore creates differences in adaptive capacity. For instance, some hunters remarked at the relative ease they had in sourcing new parts from others, either purchasing or in some cases receiving them for free with expectations of reciprocity at some point in the future. Others, citing cultural change, and the increased likelihood that other’s machines might also break, suggested that there was a lesser ethos of sharing or even selling on parts as compared with the near past, with hunters instead choosing to hold on to parts should they themselves encounter difficulties.

The points outlined above illustrate the multiscale and complex nature of potential exposure-sensitivities and adaptive capacities within the *Ulukhaktokmiut* foodshed. Critically, in order to sustain existing and develop new adaptive strategies there is a need to recognise the evident dynamism and inherent unpredictability that exists both within and between biophysical and social drivers of vulnerability. In particular, our research highlights that while a number of exogenous stressors can affect the viability of the foodshed, multiple intra-community factors—often rooted in the effects of sedentarisation—can also create significant differences in vulnerability and adaptability to climate change between community members in response to the same stimuli as a result of cascading effects. The majority of these exposure-sensitivities derive from socioeconomic factors within their familial group or social network relating to the affordability of or access to equipment and supplies. To this end, biophysical stressors, while still holding a degree of relative influence over the vulnerability of the foodshed, at present are often not the most significant determinants of foodshed vulnerability on a day-to-day basis; especially at the individual/household level. However, with the likelihood that presently infrequent anomalous extremes may become more frequent in the Arctic in coming years [3] there is the potential that new barriers to adaptation may develop in the community relating to biophysical stressors. This potential is symptomatic of a broader changing Arctic, which in an increasingly globalized world, is seeing the emergence of new stressors that have not held a similar level of precedence for a number decades (e.g. infectious diseases), or are continuing as trends with ever-increasing influence (e.g. the costs of gasoline and other supplies). This highlights the need for adaptive strategies to address complex community and individual/family-level drivers of exposure-sensitivity, particularly within socially-constructed spheres, but to also understand the greater exogenous interaction of these stimuli at regional or even global scales.

## Conclusion

This paper adopted a two-year real-time monitoring and participatory mapping methodology to examine the role of climate change as a determinant of dynamic vulnerability within a complex adaptive system: the foodshed of Ulukhaktok, NT. Our findings suggest that while environmental changes brought on by an altering climate are having a substantive impact on the stability of the *Ulukhaktokmiut* foodshed, these are often not the most salient stimuli affecting the vulnerability of the foodshed on a day-to-day basis. Instead, social drivers of vulnerability, rooted in the historical process of colonial sedentarisation (e.g. cash liquidity and access to gasoline, the time availability for hunting, and the mechanical reliability of machinery) are more immediate concerns when examining foodshed stability over the course of an entire year. In part, this may be due to the strong adaptability that *Ulukhaktokmiut* possess in the face of an incrementally changing environmental conditions, and the dual role that socioeconomic, cultural and political factors play in governing both sensitivity *and* adaptive capacity to climate change. However, it is of note that these stimuli can, and frequently do, manifest as barriers to foodshed stability almost entirely independent of climate change. Indeed, in many instances the impacts of climate change often represent an additional veneer of susceptibility that is overlain on top of a nexus of pre-existing temporally intransient stimuli in the short term. Further research in the context of climate change is necessary to develop more insights into the longer-term implications of anomalous extreme events, and how these might be shaped by further atmospheric interference. But beyond climate change, it is also evident that further exploration of the root causes of social components of vulnerability is necessary to develop more concrete understandings and to better inform adaptation that could bring about transformative change.

## Supporting information

S1 Table Supplementary quotes relating to current exposure-sensitivity and adaptive capacity in Ulukhaktok, 2018–2020. (DOCX)

S1 Fig Historic average daily ground snow thickness in Ulukhaktok as a percentage of 2018/19 values. (DOCX)
