# Supplementary material for: Monitoring the dynamic vulnerability of an Arctic subsistence food system to climate change: The case of Ulukhaktok, NT
Source: PLoS One. 2021 Sep 29;16(9):e0258048. doi: 10.1371/journal.pone.0258048 (PMC8480605; doi:10.1371/journal.pone.0258048)
Supplement: S1 Table — (DOCX) [file pone.0258048.s001.docx]

**S1 Table. Supplementary quotes relating to current exposure-sensitivity and adaptive capacity in Ulukhaktok, 2018-2020.**

| **Theme/s** | **Quote** |
| --- | --- |
| *(Climate Change)*  *Climatic variability affecting land access and safety* | “Yeah: rocks – sometimes we only go this far with skidoo. Go that way... get stuck... go backward get stuck, go forward get stuck... I was going really good [on my] last trip then, next thing I know, my skidoo was almost upside down.” (25^th^ October 2018; #043-09).  “It’s getting harder for me to predict the ice conditions now compared to back then. I can’t... I don’t even know if two and a half inches [of thickness] is good enough for me to travel on anymore.” (15th February, 2019; #057-01).  “His skidoo went through some young ice… he hit some hard-enough ice [below, rather than going into the water] and then he went back up. I don’t know what he was doing at night-time going through that stuff... I guess getting the wolf was kind of important...” (7th March, 2019; #145-12).  “We used to never use [maps], when I was growing up… we used east and west wind directions for our compass... from the east, the wind goes this way… we cut the snowdrifts if we want to go home. Now… everything changes - the wind could change anywhere.” (31st July 2019; #509-04) |
| *(Climate Change)*  *Inter-annual variations in species availability and quality, multiple exposures* | “Another muskox… this winter. It had a big ball of puss, must be about… almost the size of my fist… When we started cutting off the back for tenderloins, I thought it was a kidney problem… we took them out and in there was this big white ball of puss.” (7th July 2018; #010-02).  “They were just talking about cutting off commercial fishing [for char]… I told them just because everybody had a bad year this summer for fishing, it doesn’t mean you’ve got to panic.” (5th March 2019; #132-11).  “Hard to tell, eh, [what is happening with the char fishing]. It went down, it slowed down for a while, but looks like it’s coming back again.” (23rd July 2019; #481-09).  “Maybe 1 out of 100 I see some people tell me there’s some worms [(fish lice) (Argulus)] in the meat [of the fish]... These last two seasons, when I work [as a guide for the Department for Fisheries and Oceans], I started to note more of these worms being caught from the lake.” (18th February 2019; #100-04).  “I don’t know, there’s still some [caribou] around for sure – they’re still going to be there. I’m pretty sure they’re all coming from Hadley Bay. Coming all down, following…Kuujua, down this way…. they never eat there for like 20 years..” (3rd April, 2019; #207-17).  “When I was at the lake, one of the muskox was just… vile. [The wildlife epidemiologist] said that was the worst lungworm he’d ever seen. The whole thing… there were just lumps all over the place, and you’d cut open one and about 50, 60 or 100 worms would come out.” (7th July 2018; #010-01).  “That lungworm [we’re finding in muskox] is from a parasite… it goes from a snail into the muskox. With climate change they’ve moved north [(the parasites)]… with the weather getting warmer and the land getting greener (7th July 2018; #010-08).  My dad used to tell me “not you, not your kids, maybe not your grandkids, but one day, people are going to see trees”. (23rd July 2019; #489-05).  “When I was growing up, when I was Ben’s age we used to hear of one or two [grizzly bears] around maybe here, or there. Nowadays it’s just all over the place...” (23rd May 2019; #341-03)  “The bears that him and Edward got? They [(the bears)] killed a caribou, or a muskox each, and then only ate the foot, and some of the head too.” (May, 2019; #341-04). |
| *(Economics, Social Networks, Preparedness)*  *Mechanical issues, vehicle design* | “Me and my aluminium dog... with steel for a heart. Right now, I just threw my machine into the shop: no more bearing... driveshaft and bearing.” (7th March 2019; #161-07).  “Blew a piston! Piston had a hole right through… It was too far [gone] to fix [on the land]. I was heading back… it got hot and the oil injection I guess was not flowing or something...” (11th May, 2019; #306-13).  “Trying to get satellite signal on the phone [after I broke down], but couldn’t... I ended up walking, going up this way to get a signal somewhere. Get on top [of a hill] then I finally got a hold of [someone].” (27th March, 2019; #196-05).  “Just before I reach... near Mashuyaaq... my machine… a bolt must have came off. I went back to town, picked up another one... another machine, and took off again.” (RNK, 7th March; #125-01).  “My parents’ machine and sled box is still at Pituutaq [(out on the land)]… mine is [also] broken down. I’ve got to change the piston ring and get a new crank case [and] crankshaft! $2500... [dollars it’ll cost]… and that’s just my parent’s one!” (March 18th 2019; #215-13).  “The AR mounts broke, and the A frame broke – the one that goes to the handlebars. They were broken for a while, but they finally really broke after this rough ice!” (25th February 2019; #117-07). |
| *(Economics, Social Networks, Preparedness)*  *Cost of supplies* | “Some people used to even talk about going Kugluktuk to buy gas and bringing it over here [to store down Prince Albert Sound] for summertime. It still would have been cheaper than buying gas [in Ulukhaktok], brining it over there [to Prince Albert Sound], and then going back [to Ulukhaktok]!” (23rd July 2019; #448-16).  “People and elders… they’re always running out of naphtha. Naphtha is not cheap… 45 gallons of naphtha is 1,078 dollars, and a barrel of skidoo gas is under 400! See what the jump on that is? Triple. [Naphtha is] the main thing you use when you’re out, to keep the heat in [the tent/cabin].” (31st July 2019; #506-07). |
| *(Economics, Social Networks, Preparedness)*  *Social relationships/borrowing, adaptation, knowledge transfer, cultural change* | “For our younger generation now, without equipment they can’t go anywhere, they can’t see the land, they can’t even get food for their family” (31st July 2019; #504-08).  “Just now I need to find snowmobile parts to get my snowmobile up and running. There are lots of parts in town, it’s just that some people are too tight. And then when they come around to me, I’m not tight. You expect, you know... I scratch your back, tomorrow, or next day, or next month you scratch mine... I think that way, but then “oh, no, I’m sorry”...” (8th April 2019; #226-06).  “We never did fishing on that muskox trip, but we took fishing rods in case we didn’t see muskox.” (7th July 2018; #009-12).  “Denise was wanting to take out Peter. She was kind of iffy about letting him go, [but] she just let him go. They came back with five ptarmigan – cute kids. Really starting to travel.” (29th April 2019; #270-07).  I was trying to let Alex shoot a muskox. But the .223 [rifle]… I put a bullet in, but it couldn’t go down. I was having trouble. I grabbed Euan’s .243, but all the young ones [(muskoxen)] were all finished, so I didn’t want to try and get a big female, and I had a passenger on my sled already… None of them [(on the trip)] gave us anything [that they’d harvested]… (15^th^ February 2019; #067-16)  “He dropped all his meat. Poor tying... poor tying... you know... the rope that goes around that sled? He tied his meat down in the front [of the sled], in a tarp. There was leftover rope that was unused... He only did two ropes, and within 300 yards all his meat was falling out… He just left it there. All his meat spread out on the trail. Told him to go back for his meat: “eh, I’m just going to leave them” [he said]. He just chose not to pick it up and *payutak* [(share)], his choice... eh...” (15^th^ February, 2019; #068-13) |
| *(Institutions)*  *Harvester’s Assistance Programs, governance* | “The Nutrition North subsidy program? We said that instead of subsidizing the store-bought food they should subsidise the hunters, because the hunters spend money to get out. Not only on gas, but on vehicles; they have to repair vehicles sometimes.” (7th July 2018; #003-11).  We took students out to… Fish Lake, and then across Minto [Inlet]. Three nights, four days... and they all got muskox. There was ten students, five guys, five helpers… [we got] nineteen muskox and 41 fish! [UCC] bought the gas… and the food… I would like to see this more often: the youngest generation hunting for the community.” (29^th^ April 2019; #262-05). |
