# Supplementary material for: Monitoring the dynamic vulnerability of an Arctic subsistence food system to climate change: The case of Ulukhaktok, NT
Source: PLoS One. 2021 Sep 29;16(9):e0258048. doi: 10.1371/journal.pone.0258048 (PMC8480605; doi:10.1371/journal.pone.0258048)
Supplement: S1 Fig — (DOCX) [file pone.0258048.s002.docx]

**S1 Fig. Historic average daily ground snow thickness in Ulukhaktok as a percentage of 2018/19 values.**

| **Winter Ground Snow Thickness, Holman (Ulukhaktok) Airport Weather Station, 1^st^ Oct. – 1^st^ May** | | | |
| --- | --- | --- | --- |
| Year | Avg. daily thickness (cm) | *n* observations (of 212) | avg. daily thickness vs. 2018/19 |
| **2018/19** | **11.08** | **212 (100%)** | **-** |
| 2017/18 | 19.52 | 210 (99.1%) | 176.2% |
| 2016/17 | 23.29 | 211 (99.5%) | 210.2% |
| 2015/16 | 17.20 | 182 (85.8%) | 155.2% |
| 2014/15 | 35.54 | 169 (79.7%) | 320.8% |
| 2013/14 | 12.93 | 200 (94.3%) | 116.7% |
| 2012/13 | 18.35 | 202 (95.2%) | 165.6% |
| 2011/12 | 27.17 | 181 (85.4%) | 245.2% |
| **2010/11** | **9.18** | **181 (85.4%)** | **82.9%** |
| **2009/10** | **8.80** | **188 (88.7%)** | **79.4%** |
| 2008/09 | 13.81 | 206 (97.2%) | 124.6% |
| 2007/08 | 21.72 | 210 (99.1%) | 196% |
| 2006/07 | 22.29 | 208 (98.1%) | 201.2% |
| 2005/06 | 11.58 | 212 (100%) | 104.5% |
| 2004/05 | 18.47 | 179 (84.4%) | 166.7% |
| **All years (prior to 2018/19)** | **18.43** | **2,739 (93.2%)** | **166.3%** |

**
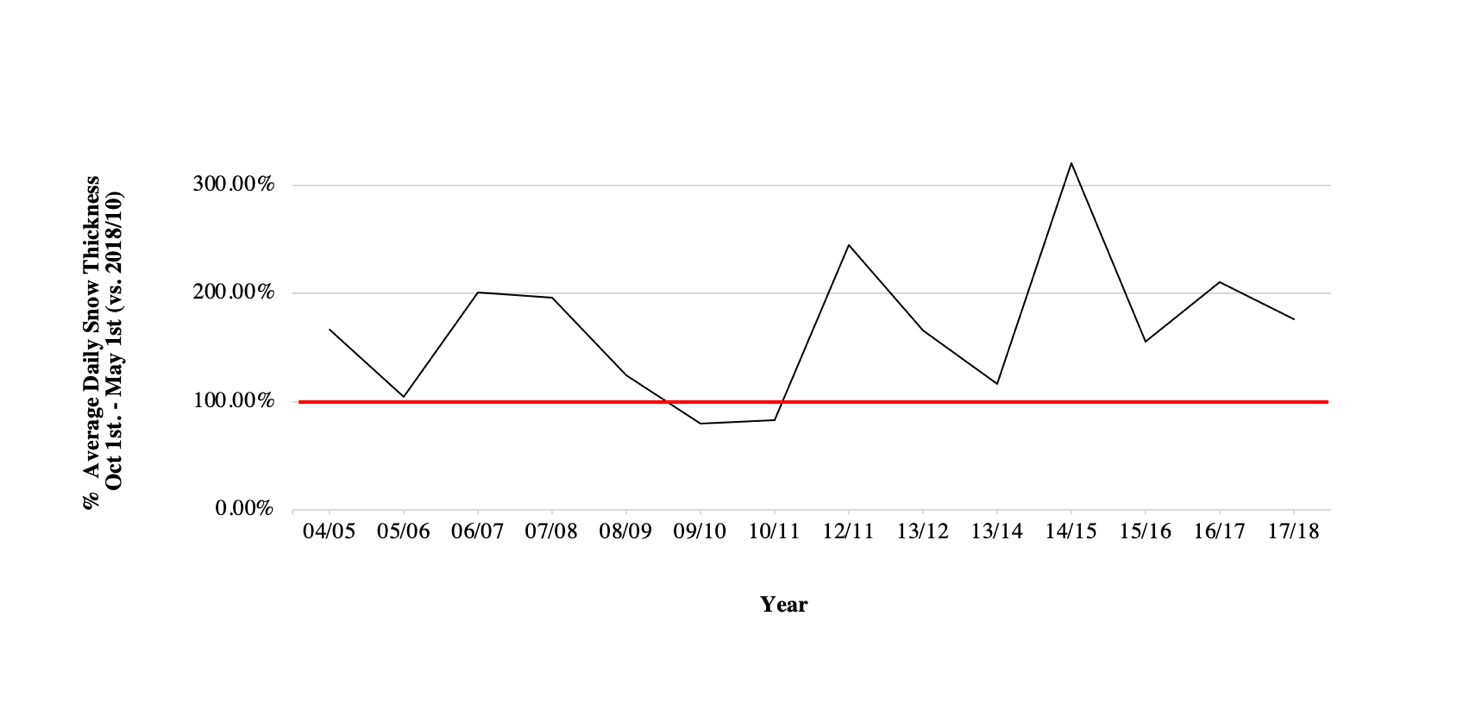
**
